# Late-life mortality is underestimated because of data errors

**DOI:** 10.1371/journal.pbio.3000148

**Published:** 2019-02-07

**Authors:** Leonid A. Gavrilov, Natalia S. Gavrilova

**Affiliations:** NORC at the University of Chicago, Chicago, Illinois, United States of America

## Abstract

Knowledge of true mortality trajectory at extreme old ages is important for biologists who test their theories of aging with demographic data. Studies using both simulation and direct age validation found that longevity records for ages 105 years and older are often incorrect and may lead to spurious mortality deceleration and mortality plateau. After age 105 years, longevity claims should be considered as extraordinary claims that require extraordinary evidence. Traditional methods of data cleaning and data quality control are just not sufficient. New, more strict methodologies of data quality control need to be developed and tested. Before this happens, all mortality estimates for ages above 105 years should be treated with caution.

## Abstract 2

This Primer explores recent evidence that demographic data errors can explain away apparent late-life mortality plateaus, proposing that traditional methods of data quality control are insufficient, and that claims of human longevity greater than 105 years should be considered extraordinary and require extraordinary evidence.

The world longevity record for Jeanne Calment (122 years) is widely cited with great pride as the gold standard of the highest data quality for many decades, not only by the Guinness Book but also in the scientific literature. Yet even for this best documented longevity claim, some early doubts were expressed of her suspicious extremely outlying age [1,2]. Biogerontologist Tom Kirkwood wrote in his book, "Could she be a fraud? It is hard to see how unless it was the mother and not the daughter who died in 1934, the daughter assuming the identity of her mother" [3]. According to one insurance book, the true Jeanne Calment died well before 1997, and her daughter assumed her identity by claiming that the daughter died instead of the mother [4]. This hypothesis explains the "perfect track record" of various administrative proofs of Jeanne's age across decades, because the identity switch would have been invisible to census and administrative officials. This "identity switch hypothesis" stimulated new studies challenging Calment’s longevity claim with a subsequent flurry of mass media coverage (S1 Text). Still, most scientists and the public believe in the validity of the Calment longevity record. The situation is even more serious—our studies found that many longevity records for ages 105 years and older are often incorrect (see later). After age 105 years, longevity claims should be considered as extraordinary claims that require extraordinary evidence. Traditional methods of data cleaning and data quality control are just not sufficient. New, more strict methodologies of data quality control need to be developed and tested. Before this happens, all mortality estimates for ages above 105 years should be treated with caution.

Knowledge of true mortality trajectory at extreme old ages is important not only for actuaries but also for biologists who test their theories of aging with demographic data. Studies conducted in the 1990s suggest that the exponential growth of human mortality with age (the Gompertz law) is followed by a period of deceleration, with slower rates of mortality increase (see Box 1). These early studies, as well as studies on insects, convinced researchers of the universality of the mortality deceleration phenomenon, and until recently, there was no doubt among biodemographers and gerontologists that mortality slows down after the age of 80 years. At that time, several biological explanations of mortality deceleration and late-life mortality plateau were suggested. Reliability models of aging also suggest mortality plateau at advanced ages when assuming random loss of functional cells and other essential elements over time [5].

**Box 1. What is mortality deceleration and mortality plateau?**

In 1825, the British actuary Benjamin Gompertz discovered a law of mortality known today as the Gompertz law [6,7,8]. Specifically, he found that the force of mortality (known in modern science as mortality rate, hazard rate, or failure rate) increases in geometrical progression with the age of adult humans. According to the Gompertz law, human mortality rates double over about every 8 years of adult age.

An exponential (Gompertzian) increase in death rates with age is observed for many biological species, including fruit flies *Drosophila melanogaster* [7], flour beetles *Tribolium confusum* [7], mice [9], rats [9], primates [10] and, perhaps most important, humans [6,7,8]. According to the Gompertz law, the logarithm of failure rates increases linearly with age. This is often used in order to illustrate graphically the validity of the Gompertz law—the data are plotted in the semilog scale (known as the Gompertz plot) to check whether the logarithm of the failure rate is indeed increasing with age in a linear fashion.

At advanced ages (after age 80 in humans), the “old-age mortality deceleration” takes place—death rates are increasing with age at a slower pace than expected from the Gompertz law.

This mortality deceleration eventually produces “late-life mortality leveling-off” and “late-life mortality plateaus'” at extreme old ages [7,8,11,12]. Gompertz himself first noted this phenomenon, and later actuaries proposed a logistic formula for mortality growth with age in order to account for mortality deceleration at advanced ages. Greenwood and Irwin were the first who provided a detailed description of this phenomenon for humans and even made the first estimates for the asymptotic value of human mortality [12]. According to their estimates, the mortality kinetics of long-lived individuals is close to the law of radioactive decay with half-time approximately equal to one year.

The same phenomenon of “almost nonaging” survival dynamics at extreme old ages is detected in other biological species. In some species (medflies and house flies), mortality plateau can occupy a sizable part of their life [5,13]. In the 1990s, the phenomenon of mortality deceleration and leveling-off became widely known after publications that demonstrated mortality leveling-off in large samples of fruit flies [11] and medflies [13]. The existence of mortality plateaus is now well documented for a number of lower organisms, including medfly [13], house fly *Musca domestica* [5], fruit flies *Anastrepha ludens*, *A*. *obliqua*, *A*. *serpentine*, and parasitoid wasp *Diachasmimorpha longiacaudtis* [11,14]. In the case of mammalian species, the situation is more controversial, and it seems that, for primates [10], mice, and rats [9], no mortality deceleration can be found.

An immediate consequence from the mortality deceleration phenomenon is that there is no fixed upper limit to human longevity—there is no special fixed number that separates possible and impossible values of lifespan [7,15]. This conclusion is important, because it challenges the common belief in the existence of a fixed maximal human lifespan and biological limit to longevity.

However, new results discussed in this article challenge the existence of late-life mortality plateau in humans when data are of better quality.

Recently, the common view about mortality deceleration at advanced ages has been challenged using both theoretical [16] and empirical [9,17] considerations. It was found that mortality of US extinct cohorts born after 1889 demonstrated the Gompertz-like trajectory in the age interval 85 to 106 years [9,17]. In the study of old-age mortality in 15 low-mortality countries, Bebbington and colleagues found the Gompertz-like mortality growth at older ages for Australia, Canada, and the US and mortality deceleration for other studied countries [18].

It should be noted that hazard rate estimation at very old ages faces difficulties because of very small number of survivors to these ages, and age misreporting by older persons. Age misreporting is a big problem affecting estimates of mortality at advanced ages [19,20]. It was found that even a small percentage of inaccurate data can greatly distort mortality trajectories at advanced ages [20,21] and that age misreporting at older ages results in mortality underestimation [19]. Taking into account that the accuracy of age reporting is positively correlated with education [22], it is reasonable to expect improvement in age reporting over time and less prevalent mortality underestimation or mortality deceleration at older ages for more recent birth cohorts. Indeed, it was found that late-life mortality in historically older US birth cohorts demonstrates stronger mortality deceleration compared to more recent birth cohorts [17]. These results suggest that mortality deceleration observed in early studies of old-age mortality may be caused by age misreporting at older ages [23,24].

Newman demonstrates that rare cases of age misreporting among people in their eighties eventually result in accumulation of false claims at extreme old ages and produce spurious mortality deceleration [25,26]. Regardless of possible limitations of his simulation model (as Wachter [27] rightfully pointed to significant age exaggeration in Newman’s model), this study is important because it brings our attention to the fact that even rare cases of age misreporting are able to produce spurious mortality deceleration. Currently, information about quality of age reporting at extreme old ages is hardly ideal even in countries with good vital statistics. Italian data used by Barbi and colleagues [28] have apparently a good quality for ages 110 years and over, because these records were validated using birth certificates [27]. However, for ages 105 to 109 years, the quality of data remains uncertain because the authors do not outline in detail what they did to verify and clean the data and what is the percentage of incorrect age reporting. Wachter suggests that age misreporting in data used by Barbi and colleagues should be minimal because Italy has a well-established registration system [27]. But are Italian data really free of errors? If we look at Fig 1 in Barbi’s paper, then we can see a drop of mortality at age 100 years, which is most likely a consequence of age heaping when nonagenarians round their age to 100 [28]. This example shows us that age misreporting may affect mortality estimates. Taking into account that data errors have a tendency to accumulate with age [29,30], it would be reasonable to expect more errors at ages 105 years and over.

**Fig 1:**
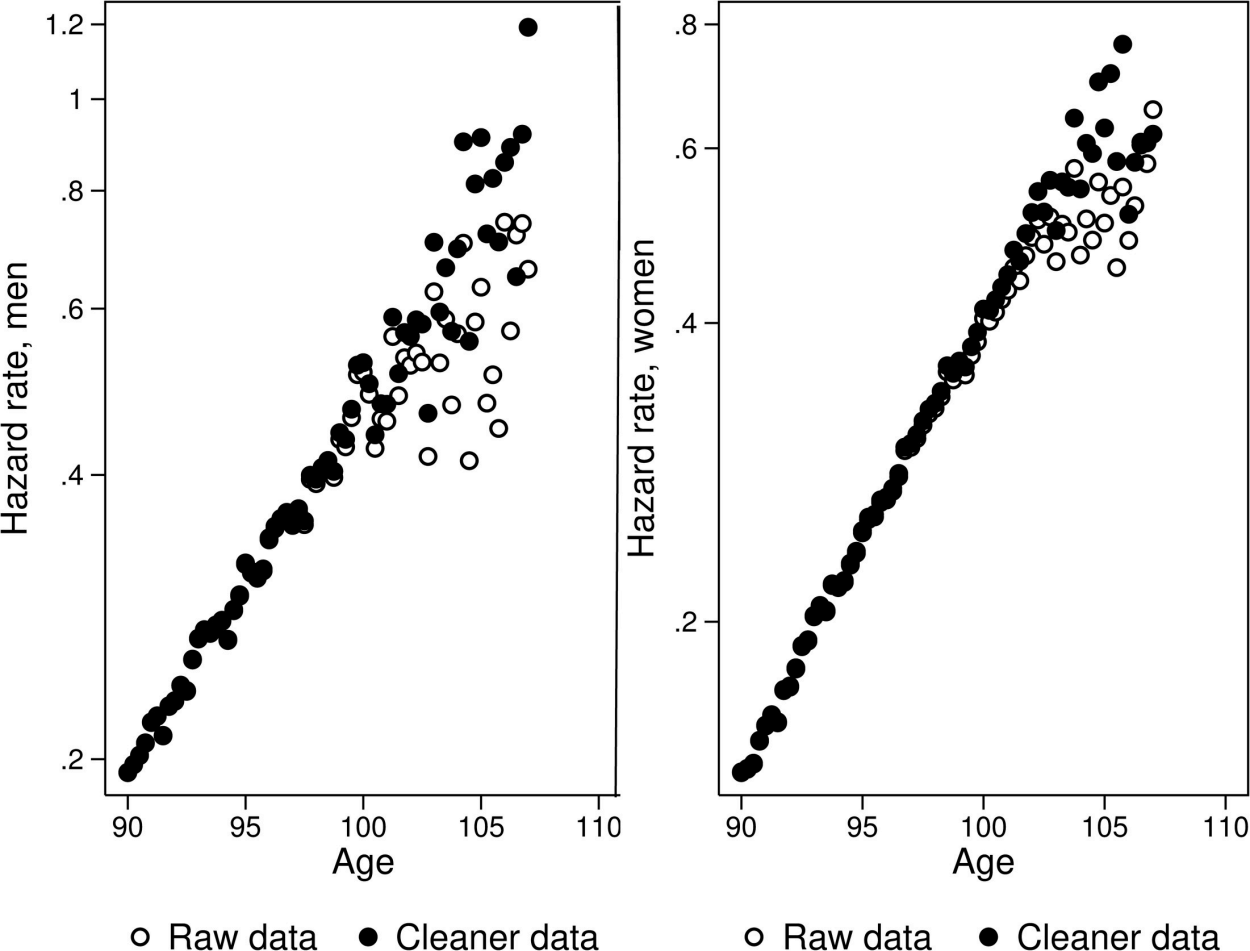
Data cleaning increases late-life mortality estimates. Mortality of U.S. men (on the left) and women (on the right) born in 1900 as a function of age, by the degree of data quality (S1 Data).

According to our experience, age misreporting indeed is able to produce false mortality deceleration. We conducted a direct age validation for records of persons born in 1900 and aged 106 years and over using data available in the US Social Security Administration Death Master File (DMF; S2 Text). During this age validation procedure, records for persons aged 106 years and older in the DMF were searched and linked to historical resources available in Ancestry.com (early US censuses, birth certificates, military records, etc.) in order to confirm the age indicated in the DMF database. Overall, 930 records were checked. It turns out that the proportion of records with unconfirmed age for US persons born in 1900 increases from 12% at age 100 years to 17% at age 105 years and 35% at ages 109 years and over [30]. Because the US has a large population size, the advantage of US data is a significant number of survivors to advanced ages allowing researchers to obtain more reliable estimates for hazard rate.

Fig 1 shows the effect of data cleaning after age 105 years on mortality trajectories at extreme old ages for US men and women. Hazard rate is measured using quarterly (3-month) age intervals expressed in yearly units (1/year). Use of more detailed quarterly age intervals allows us to obtain more accurate estimates of hazard rate and avoid potential spurious mortality deceleration [17]. Fig 1 demonstrates that improvement of data quality (data cleaning when only persons with confirmed age at death are included) changes the shape of trajectories from mortality plateau to mortality growth with age. This figure also does not demonstrate any noticeable mortality deceleration between ages 95 and 105 years.

It should be noted that mortality after age 105 years is subject to significant variability, so it is often difficult to select a specific model for mortality trajectory. However, in most cases, mortality demonstrates a growing pattern. Difficulty in getting a definite conclusion about the model of mortality after age 105 years can be seen from the results by Barbi and colleagues [28]. Their comparison of constant mortality model and the Gompertz model produced a very small difference in the Akaike Information Criterion (AIC) (AIC difference equal to 1.49 only), suggesting very weak support of constant mortality model by data. Support by data is considered to be strong if the AIC difference is at least 10 or higher [31]. It would be interesting to reanalyze mortality trajectory specifically for the1896 to 1901 extinct birth cohort, because this cohort contains the most records with verified 110 years and older ages and hence has better quality. Our prediction is that in this particular birth cohort, the evidence for mortality plateau will be even weaker.

Italian data are unusual in the sense that they demonstrate a historical decline of mortality even at very advanced ages of 105 years and over [28]. A recent observation in many countries is that mortality of centenarians did not decrease noticeably in the past decade, despite a significant decline in mortality of younger age groups [32,33]. Historical stagnation of mortality at ages 100 years and older may lead to steeper mortality curves for cohorts at extreme old ages, whereas historical mortality decline at old ages may produce an apparent decelerating pattern of mortality with age. Feehan fitted old-age mortality for cohorts in different countries using several competing models [34]. He found that the Gompertz model performed poorly for France and Italy, whereas for Sweden and the Netherlands this model performed reasonably well. He suggested that period effects may have altered the shape of cohort mortality producing the strongest evidence of non-Gompertz death rates for cohorts that experienced continuous historical improvements (like France and Italy). Therefore, he cautioned not to make sweeping generalizations about the shape of mortality trajectories at advanced ages based on results obtained in one country [34]. In view of this, it appears that the late-life mortality plateau should not be considered as a universal biological law of mortality.

For biologists, problems with data quality at advanced ages open a new avenue of research to develop more accurate methodology of calendar age estimation for very old people. Current methods of age estimation used in forensic medicine may work well for younger people, whereas in the case of extremely old individuals, these methods may not be sufficiently accurate. This is a great challenge (because we are not trees and do not have annual rings) and opportunity for future research. As for the world longevity record by Jeanne Calment, the identity switch hypothesis could be decisively resolved by DNA testing.

## Supporting information

S1 Text List of new papers, internet resources and media coverage challenging the validity of Jeanne Calment record. (PDF)

S2 Text Description of data and methods underlying Fig 1. (PDF)

S1 Data Data underlying Fig 1. (XLSX)

## Abbreviations

AIC: Akaike Information Criterion
DMF: Death Master File
