# Supplementary material for: Late-life mortality is underestimated because of data errors
Source: PLoS Biol. 2019 Feb 7;17(2):e3000148. doi: 10.1371/journal.pbio.3000148 (PMC6386419; doi:10.1371/journal.pbio.3000148)
Supplement: S1 Text — (PDF) [file pbio.3000148.s001.pdf]

S1. List of new papers, internet resources and media coverage challenging the validity of Jeanne Calment record.

New paper challenging the Jeanne Calment longevity record:

Zak, N. Evidence that Jeanne Calment died in 1934, not 1997. Rejuvenation Research. Published Online:30 Jan 2019. https://doi.org/10.1089/rej.2018.2167

New online publications and preprints challenging the 122-year longevity record of Jeanne Calment:

Deigin, Yuri (10 December 2018). J'Accuse…! Why Jeanne Calment's 122-year old longevity record may be fake. Retrieved 12 December 2018 – via Medium. https://medium.com/@yurideigin/jaccuse-why-122-year-longevity-record-may-be-fake-af87fc0c3133

Deigin, Yuri (25 December 2018). More evidence for Jeanne Calment's identity theft hypothesis. Retrieved 30 December 2018 – via Medium. https://medium.com/@yurideigin/more-evidence-for-jeanne-calments-identity-theft-hypothesis-26f7cece0cd2

Deigin, Yuri (6 January 2019). Oh Jeanne, why do you look so young? I mean, so old. Wait, which one is it? Retrieved 11 January 2019 – via Medium. https://medium.com/@yurideigin/oh-jeanne-why-so-young-8e8019967bfc

Deigin, Yuri. Jeanne and Me, (16 January, 2019). The story of my participation in the Jeanne Calment investigation. Retrieved February 1, 2019. https://medium.com/@yurideigin/jeanne-and-me-7d0f04314acb

Zak, Nikolay (December 2018). Jeanne Calment: the secret of longevity. ResearchGate. doi:10.13140/RG.2.2.29345.04964. Retrieved 27 December 2018. https://www.researchgate.net/publication/329773795_Jeanne_Calment_the_secret_of_longevity

Media coverage of new studies challenging Jeanne Calment longevity record:

Oldest ever woman Jeanne Calment, 122, may have been a fraud. Adam Sage, *The Times*. London, 1 January 2019. Retrieved 1 January 2019. https://www.thetimes.co.uk/article/woman-lived-as-122-year-old-mother-to-dodge-tax-cfwd8bcm6

Was the World's Oldest Person Ever Actually Her 99-Year-Old Daughter? Jason Daley, *Smithsonian magazine*, (January 2, 2019). Retrieved 3 January, 2019. https://www.smithsonianmag.com/smart-news/study-questions-age-worlds-oldest-woman-180971153/

The world's oldest person record stood for decades. Then came a Russian conspiracy theory. Eli Rosenberg, *Washington Post*, January 12, 2019. Retrieved 14 January 2019. https://www.washingtonpost.com/world/2019/01/12/how-madame-calment-worlds-oldest-person-became-fuel-russian-conspiracy-theory

History's oldest woman a fraud? Russian researchers claim 122-year-old Jeanne Calment was actually a 99-year-old imposter. Tristin Hopper, *National Post*. Toronto, Canada, 31 December 2018. Retrieved 1 January 2019. https://nationalpost.com/news/world/historys-oldest-woman-a-fraud-theory-says-122-year-old-jeanne-calment-was-actually-a-99-year-old-imposter

World's oldest-ever person was fraud who stole mother's identity, researchers claim. Tom Barnes, *The Independent*, UK, January 2, 2019. Retrieved 3 January 2019. https://www.independent.co.uk/news/world/europe/world-oldest-person-ever-fraud-scam-identity-theft-jeanne-calment-mother-conspiracy-a8708271.html

Onetime world's oldest woman may have lied about her age. Lia Eustachewich, *New York Post*, January 2, 2019. Retrieved 3 January 2019. https://nypost.com/2019/01/02/onetime-worlds-oldest-woman-may-have-lied-about-her-age/

Report claims fraud in Frenchwoman's 'oldest-ever' world record. Victoria Loguinova-Yakovleva, *Yahoo! News*, January 2, 2019. Retrieved 3 January 2019. https://news.yahoo.com/report-claims-fraud-frenchwomans-oldest-ever-world-record-112541093.html

Researchers claim world record for longest life a case of ID fraud. *CBS News*, January 2, 2019. Retrieved 3 January 2019. https://www.cbsnews.com/news/worlds-longest-living-person-jeanne-calment-fraud-russian-researcher-guinness-record/

World's 'oldest woman' who 'died at 122' may actually have been her 100-year-old daughter, researchers claim. Jonathan Reilly, *The Sun*, January 2, 2019. Retrieved 3 January 2019. https://www.thesun.co.uk/news/8100298/worlds-oldest-woman-122-imposter/

World's Oldest Person, Jeanne Calment, May Have Faked Her Age: Report. David Moye, *Huffington Post*, January 1, 2019. Retrieved 2 January 2019. https://www.huffingtonpost.com/entry/jeanne-calment-worlds-oldest-person-faked-age_us_5c2bb81fe4b05c88b703705e

World's oldest person ever, Jeanne Calment, may have been a fraud, researchers allege. Kathleen Joyce, *Fox News*, January 1, 2019. Retrieved 1 January 2019. https://www.foxnews.com/world/worlds-oldest-person-ever-jeanne-calment-may-have-been-a-fraud-researchers-allege
