# Supplementary material for: Late-life mortality is underestimated because of data errors
Source: PLoS Biol. 2019 Feb 7;17(2):e3000148. doi: 10.1371/journal.pbio.3000148 (PMC6386419; doi:10.1371/journal.pbio.3000148)
Supplement: S2 Text — (PDF) [file pbio.3000148.s002.pdf]

S2. Data and Methods

Data. Information on U.S. mortality was taken from the Social Security Administration Death Master File (DMF) or the Social Security Death Index (SSDI), which was obtained from the U.S. National Technical Information Service (NTIS) in September 2011. Currently SSDI is available at Ancestry.com for deaths occurred between 1935 and 2014. Taking into account that the latest deaths in our version of DMF were observed in September 2011, additional information on deaths occurring in later years was taken from the Ancestry.com SSDI database and the Gerontology Research Group Table A (available at http://gerontology.wikia.com/wiki/List_of_supercentenarians_born_in_1900).

Quality control procedure. Age validation for ages 106+ years consisted of direct linking individual records of old individuals to historical resources available at Ancestry.com. This procedure resulted in setting a quality score for each record. The scoring system used in this study is described in the following report: Gavrilova NS, Gavrilov LA (2018) Mortality Analysis of 1898-1902 Birth Cohort. Shaumburg, IL: Society of Actuaries.

Hazard rate estimation.Hazard rates were estimated using the Stata statistical package (release 14), procedure *ltable*. Procedure *ltable* calculates hazard rate for discrete data in the following way. Let $f_j = d_j/n_j$ be within-interval failure rate, where d_j is number of deaths within interval *j* and n_j is number alive at the beginning of interval *j*. Then the maximum likelihood estimate of the force of mortality (hazard rate) for interval *j* is:

$$\square_j = \frac{1}{\square x} \frac{f_j}{1 - \frac{f_j}{2}}$$

where Δx is the length of age interval *j*.

This estimate of hazard rate is also called an actuarial estimate or age-specific death rate. Its main assumption is uniform distribution of deaths in the age interval. This empirical estimate provides nonbiased estimates of hazard rate at old ages (up to age 110 years) in contrast to Nelson-Aalen yearly estimate, which has a theoretical upper boundary equal to one.
